# Key factors influencing medical residency choice among medical graduates

**DOI:** 10.1186/s13584-026-00768-x

**Published:** 2026-06-17

**Authors:** Reut Shoham, Mor Vaknin, Yuval Tal, Nevo Yahav, Arnon Afek, Gad Segal, Shalev Fried, Ofira Zloto

**Affiliations:** 1 Education Authority, Sheba Medical Center, Tel Hashomer, Ramat Gan, Israel; 2 Gray Faculty of Health Sciences and Medicine, Tel Aviv University, Tel Aviv, Israel; 3 The Goldschleger Eye Institute, Sheba Medical Center, Tel-Hashomer, Israel; 4 Recanati School of Medicine, Reichman University, Herzliya, Israel; 5 Gertner Institute for Epidemiology and Health Policy Research, Sheba Medical Center, Ramat Gan, Israel

**Keywords:** Specialty choice, Medical students, Interns, Exploratory factor analysis, Motivational factors, Workforce planning, Lifestyle considerations, Career and academic development

## Abstract

**Background:**

Specialty choice plays a central role in shaping physicians’ careers and influences the distribution and sustainability of the healthcare workforce. In Israel, persistent shortages in specific specialties and geographic regions highlight the need to better understand the motivations guiding specialty preferences. Identifying latent motivational dimensions provides a broad perspective on the interplay between multidimensional factors shaping specialty choice.

**Methods:**

A cross-sectional survey was conducted among Israeli medical students in their final year and medical interns between July 2024 and January 2025. Participants completed an online questionnaire including demographic characteristics and motivational factors influencing specialty choice using 5-point Likert-scale items. Exploratory factor analysis based on polychoric correlations was performed to identify latent motivational dimensions. Factor associations with demographic characteristics and intended specialty were examined using proportional-odds ordinal logistic regression.

**Results:**

A total of 527 trainees participated. Exploratory factor analysis identified three motivational dimensions: Interpersonal Factors, Career and Academic Development, and Lifestyle Aspects. All factors demonstrated acceptable internal consistency. No sex-based differences were observed for lifestyle or career and academic motivations, while women rated interpersonal considerations higher than men. Career and academic development motivations were stronger among trainees who studied abroad and among graduates compared with final-year students. Specialty-specific analyses showed that candidates intending surgical or obstetrics and gynecology specialties placed the greatest emphasis on career and academic development, while lifestyle considerations were more prominent among those aiming for pediatrics and psychiatry. Interpersonal motivations did not differ significantly across specialties.

**Conclusions:**

Specialty choice among Israeli medical trainees is shaped by distinct but interrelated motivational dimensions with important workforce implications. The shared importance of lifestyle considerations across sexes suggests that improvements in work–life balance may enhance recruitment across a wider range of specialties. Specialty-specific differences in career and academic motivations highlight the potential value of structured exposure during medical school rotations to research activity and academic career pathways, particularly in fields perceived as offering limited academic opportunities. Aligning training environments with trainee motivations may support recruitment to specialties in distress and contribute to long-term workforce sustainability.

**Supplementary Information:**

The online version contains supplementary material available at 10.1186/s13584-026-00768-x.

## Background

Choosing a residency specialty is a pivotal decision that shapes physicians’ career trajectories, future satisfaction, and long-term professional path. Specialty choices are influenced by multiple interacting factors including personality traits, financial considerations, lifestyle expectations, educational exposure, and demographic characteristics [1–5]. These decisions are made sometimes with incomplete or indirect information about the realities of different fields, which means that perceptions of lifestyle or clinical roles may not always align with actual practice [6–9]. Specialty choices carry important system-level implications, as they influence the distribution of specialists and the long-term sustainability of the healthcare workforce.

Global workforce projections highlight growing physician shortages in developed countries, particularly in primary care and remotely located regions. Israel faces similar challenges. Although its physician-to-population ratio is close to the OECD average, substantial variability exists across different specialties and geographic areas [10]. Understanding what drives specialty preferences is essential for anticipating workforce gaps and supporting policy interventions.

Previous research has examined factors influencing specialty choice mainly through descriptive or correlational methods. Exploratory factor analysis (EFA) identifies latent motivational dimensions that integrate related variables for a more comprehensive understanding of career decision-making [11]. This study applies EFA to examine the underlying motivational factors guiding Israeli medical interns in their specialty choices, offering insights to support workforce planning and system-level optimization.

## Methods

This study included Israeli medical students who were either in their internship year or in the final year of their medical education. Participants were recruited between July 2024 and January 2025 through invitations posted in Facebook groups, student communities, and WhatsApp messaging groups. Eligible individuals were medical students of any age, sex, or ethnic background who were completing the final year of an accredited medical program in Israel or abroad, as well as interns training in Israeli medical centers. Students who completed the questionnaire were entered into a raffle for a laptop computer. The study protocol was approved by the Sheba Medical Center Institutional Review Board (SMC-24-0968) and adhered to the principles of the Declaration of Helsinki. The first item in the questionnaire was a consent to participate in the study.

### Study questionnaire

An online questionnaire was created for this study (Supplementary Material 1). It included multiple-choice demographic questions and a series of items rated on a 5-point Likert scale assessing the influence of various factors on residency specialty selection, with responses ranging from 1 (“strongly disagree”) to 5 (“strongly agree”). This online questionnaire was developed for this study by a multidisciplinary team comprising senior medical education administrators, clinicians, and organizational social psychologists. The instrument was developed based on a comprehensive literature review of medical specialty choice and was structurally adapted from established international and national frameworks, including prior validated Israeli medical workforce surveys. To ensure content validity and cultural adaptation to the unique structure of the Israeli residency match system, an initial pool of items was evaluated by an expert panel. Items were refined to capture the contemporary challenges of Israeli trainees, such as older age at graduation and family commitments.

### Data analysis

All analyses were conducted in Python 3.12.10, with exploratory factor analysis performed in R 4.5.1 via Python bridge. By integrating these environments, we utilized a computational framework that allowed for the implementation of advanced ordinal modeling techniques often absent from the default menus of standard statistical software.

Data suitability was assessed using sample size considerations, the Kaiser–Meyer–Olkin (KMO) measure of sampling adequacy, and Bartlett’s test. While Pearson correlations are the common default for factor analysis of Likert scale data, they can artificially restrict variability and underestimate associations when applied to ordinal measures. Consequently, a polychoric correlation matrix was utilized to respect the ordinal nature of the variables, providing a more robust and accurate model [12].

Factor retention was guided by parallel analysis, scree plot, and eigenvalues. Items with loadings above 0.40 and communalities above 0.20 were retained, and each factor included at least three items. Internal consistency was evaluated using Cronbach’s α based on polychoric correlations.

Associations between participant characteristics and factor scores were examined with proportional-odds ordinal logistic regression using tertiles of each factor score as outcomes.

Predictors included sex, parental and marital status, study stage, age, and medical school location. A second set of models assessed associations between motivational factors and intended specialty (grouped into surgical, internal, pediatrics, obstetrics and gynecology, and psychiatry, see Supplementary Material 2 for full specialty classifications). The second regression included demographic, educational covariates, and pairwise contrasts between specialty groups. Benjamini–Hochberg FDR correction was applied to the specialty models. Results are reported as odds ratios with 95% confidence intervals and, where applicable, FDR adjusted p-values. Only comparisons that remained statistically significant after FDR adjustment were reported.

## Results

### Study population

A total of 527 trainees participated in the study, including 285 (54.1%) women and 242 men. According to the Israeli Ministry of Health internship lottery data, our potential cohort included a total of 4,520 trainees (1,593 Israel trained and 2,927 foreign trained) who participated in the 2024 and 2025 lotteries, corresponding to an overall response rate of approximately 11.7%, which is within a range previously shown to yield reliable estimates in large-sample survey studies [13]. The average age was 30.5 years (SD 3.4). Most respondents were married or in a long-term relationship (61.5%), and 30.7% had children. The majority rated their socioeconomic status as average (62.9%). Most participants were born in Israel (85.8%), and Jewish respondents comprised the majority of the cohort. Table 1 presents the baseline characteristics of the study population.

**Table 1:** Participants’ characteristics

Characteristic	*n* = 527
Sex (Female)	285 (54.1%)
Age (mean ± SD)	30.5 ± 3.4
*Family status*	
Married/Long - term relationship	323 (61.5%)
Single	196 (37.3%)
Divorced	6 (1.1%)
Have children	161 (30.7%)
*Socioeconomic status*	
Above average	87 (16.6%)
Average	329 (62.9%)
Below average	107 (20.5%)
*Medical school*	
Israel	382 (72.9%)
Abroad	142 (27.1%)
*Training stage*	
Final year of medical school	303 (57.7%)
Intern	119 (22.7%)
Graduated, internship not started	103 (19.6%)
*Intended Specialty*	
Surgical specialties	141 (26.9%)
Pediatrics	87 (16.6%)
Internal specialties	86 (16.3%)
Obstetrics and Gynecology	66 (12.6%)
Psychiatry (Adult/Child)	39 (7.4%)
Other	106 (20.2%)

### Factor analysis

An EFA of the 19 Likert-type motivational items yielded a three-factor solution that met all extraction and interpretability criteria. Data adequacy was confirmed by strong sampling indicators (*n* = 516; N: p ratio = 27:1; KMO = 0.78) and a significant Bartlett’s test of sphericity (χ² = 4115.20, *p* < 0.001). Parallel analysis suggested a five-factor solution. However, inspection of this solution showed limited interpretability, including suboptimal internal consistency (Cronbach’s α < 0.70 for two factors) and the presence of a factor defined by fewer than three items. In contrast, the three-factor solution showed a stable structure with clearly defined factors and adequate internal consistency and was therefore retained. Three items (interest in a specific disease, difficulty coping with death, and occupational risks) showed weak and diffuse loadings across factors and were therefore excluded. The remaining 16 items loaded clearly (≥ 0.40) on a single factor, with no cross-loadings ≥ 0.40. Factor loadings are shown in Table 2.

**Table 2:** Factor loadings in polychoric exploratory factor analysis

	F1	F2	F3	Primary factor (|loading|≥0.40)	Primary loading
Promotion opportunities	0.06	0.59	0.01	F2	0.59
Interest in procedures	-0.03	0.49	0.02	F2	0.49
Interest in specific disease	0.29	0.34	0.09	—	
Income potential	-0.17	0.35	0.63	F3	0.63
Work–life balance	0.04	-0.23	0.79	F3	0.79
Private practice opportunity	-0.13	0.17	0.53	F3	0.53
Direct patient care	0.74	-0.26	0.00	F1	0.74
Work environment	0.50	0.06	0.22	F1	0.50
Working hours	0.12	-0.18	0.71	F3	0.71
Research opportunities	0.11	0.69	-0.19	F2	0.69
Technology integration	-0.08	0.72	0.10	F2	0.72
Academic promotion	0.11	0.75	-0.07	F2	0.75
Difficulty coping with death	0.28	0.26	0.38	—	
Sense of purpose	0.69	0.06	-0.13	F1	0.69
Teamwork	0.64	0.22	-0.03	F1	0.64
High quality teaching	0.61	0.32	0.03	F1	0.61
Patient communication quality	0.79	0.00	0.06	F1	0.79
Occupational risks	0.08	0.30	0.40	—	
Difficulty getting residency position	-0.03	0.24	0.47	F3	0.47

The three retained factors accounted for 44.8% of the total variance (Factor 1 = 15.9%, Factor 2 = 15.9%, Factor 3 = 12.9%). Based on the content and loading patterns of the retained items, three distinct motivational dimensions were identified (Table 3).

**Table 3:** Item composition of the three motivational factors identified in the EFA

F1 Interpersonal Factors	F2 Career & Academic Development	F3 Lifestyle Aspects
Direct patient care	Promotion opportunities	Income potential
Work environment	Interest in procedures	Work–life balance
Sense of purpose	Research opportunities	Private practice opportunity
Teamwork	Technology integration	Working hours
High quality teaching	Academic promotion	Difficulty getting residency position
Patient communication quality		

All factors demonstrated acceptable internal consistency, with Cronbach’s α values of 0.83, 0.79, and 0.75 for F1, F2, and F3, respectively, indicating that the items within each factor were strongly interrelated and consistently reflected the same underlying motivational dimension.

### Logistic regression

#### Demographic and educational predictors

A multivariable ordinal logistic regression examined the associations between the three motivational factors and participants’ demographic and educational characteristics (Table 4). Female participants rated interpersonal aspects higher than males (OR = 2.14, 95% CI 1.53–3.01, *p* < 0.005). No significant associations were observed for age, marital status, or parental status. Among educational variables, both training stage and medical school location (Israel vs. Abroad) were associated with career and academic development motivation. Interns or recent graduates prioritized career-related aspects more than medical students in their final year (OR = 1.58, 95% CI 1.13–2.22, *p* = 0.008). Participants who studied abroad placed greater emphasis on career-related considerations than those who studied in Israel (OR = 3.29, 95% CI 2.16–4.99, *p* < 0.005) and also rated lifestyle factors higher (OR = 1.93, 95% CI 1.29–2.87, *p* < 0.005).

#### Specialty-based predictors

A second set of multivariable ordinal logistic regressions was performed among participants who intended to pursue one of the main specialty groups (Internal, Surgical, Pediatrics, Obstetrics and Gynecology, or Psychiatry), adjusting for demographic and educational characteristics and applying false discovery rate (FDR) correction (Table 5).

**Fig. 1:**
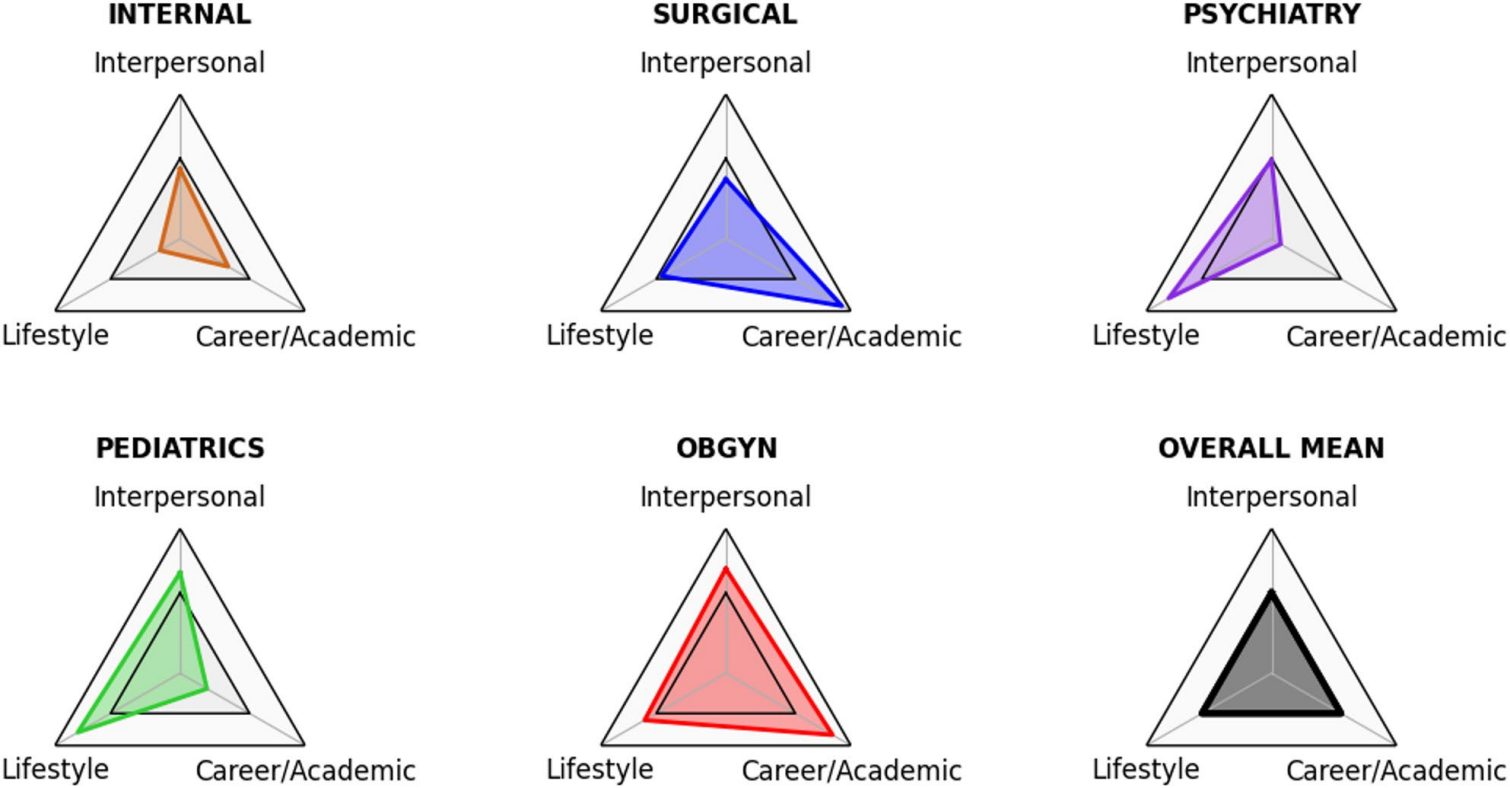
Motivational factors profile by specialty. Each panel shows a specialty’s mean z-scores for the three latent motivational factors. The shaded triangle represents that specialty’s factor importance profile, while the inner triangle marks the sample mean, and the outer triangle marks the observed range

Participants aspiring to Surgical specialties (OR = 2.55, 95% CI 1.43–4.53, *p* = 0.015) and Obstetrics and Gynecology (OR = 2.57, 95% CI 1.33–4.95, *p* = 0.025) assigned significantly greater importance to career and academic development compared with those intending to pursue Pediatrics. Lifestyle considerations were ranked higher among those aiming for Psychiatry (OR = 3.55, 95% CI 1.68–7.51, *p* = 0.009) and Pediatrics (OR = 2.30, 95% CI 1.30–4.07, *p* = 0.021) compared with Internal specialties. Furthermore, candidates for Psychiatry prioritized lifestyle factors significantly more than their peers in Surgical specialties (OR = 2.62, 95% CI 1.26–5.46, *p* = 0.034). Interpersonal considerations did not differ significantly across specialties. Figure 1 displays the motivational profiles of each specialty group compared to the overall sample.

**Table 4:** Demographic and educational predictors of interpersonal, career/academic, and lifestyle motivation factors.

Factor	F1 (Interpersonal)		F2 (Career/Academic)		F3 (Lifestyle)	
	OR [95%CI]	*p*-value	OR [95%CI]	*p*-value	OR [95%CI]	*p*-value
**Female vs. Male**	**2.14 [1.53–3.01]**	**< 0.005**	1.19 [0.85–1.67]	NS	1.06 [0.77–1.48]	NS
**Has kids vs. No kids**	1.27 [0.83–1.92]	NS	0.86 [0.57–1.31]	NS	1.17 [0.77–1.77]	NS
**Married vs. Single/Divorced**	0.75 [0.50–1.11]	NS	0.68 [0.46–1.02]	NS	1.09 [0.73–1.62]	NS
**Graduate vs. final year**	1.10 [0.79–1.54]	NS	**1.58 [1.13–2.22]**	**0.008**	0.95 [0.68–1.33]	NS
**Age (≥ 30 vs. < 30 )**	1.31 [0.92–1.86]	NS	1.07 [0.75–1.52]	NS	0.94 [0.66–1.34]	NS
**Med school: Abroad vs. Israel**	1.07 [0.72–1.58]	NS	**3.29 [2.16–4.99]**	**< 0.005**	**1.93 [1.29–2.87]**	**< 0.005**

Bold value indicates a statistically significant difference.

**Table 5:** Pairwise specialty comparisons for career/academic and lifestyle motivation factors.

Factor	Group 1	Group 2	OR [95% CI]	*p*-value
**Career/Academic**	Surgical specialties	Pediatrics	2.55 [1.43–4.53]	**0.015**
	OBGYN	Pediatrics	2.57 [1.33–4.95]	**0.025**
**Lifestyle**	Psychiatry (Adult/Child)	Internal specialties	3.55 [1.68–7.51]	**0.009**
	Pediatrics	Internal specialties	2.30 [1.30–4.07]	**0.021**
	Psychiatry (Adult/Child)	Surgical specialties	2.62 [1.26–5.46]	**0.034**

Bold value indicates a statistically significant difference.

## Discussion

This study identified three latent dimensions underlying specialty preferences among Israeli medical students and graduates: Interpersonal Factors (F1), Career and Academic Development (F2), and Lifestyle Aspects (F3). Compared with men, women assigned higher scores to interpersonal considerations. Participants who trained abroad, relative to those trained in Israel, reported stronger career- and lifestyle-related motivations. Graduates (interns or pre-internship) also placed greater emphasis on career and academic development than students in their final year. Specialty comparisons showed that career-oriented factors were most influential among those pursuing surgery, whereas lifestyle considerations were stronger among those choosing psychiatry or pediatrics.

To move beyond purely descriptive categorizations, these three empirically derived factors can be meaningfully interpreted through the lens of Self-Determination Theory [14], which posits that human motivation and optimal functioning are driven by the fulfillment of three basic psychological needs: autonomy, competence, and relatedness.

Our Interpersonal Factors (F1) dimension maps directly onto the need for *Relatedness*, the intrinsic desire to feel connected to others, engage in meaningful patient communication, and experience cohesive teamwork. The Career and Academic Development (F2) factor aligns with the need for *Competence*, capturing trainees’ aspirations to master specialized procedural skills, achieve academic promotion, and integrate advanced technologies. Finally, the emergence of Lifestyle Aspects (F3) reflects a modern evolution of the need for *Autonomy* and well-being, where contemporary trainees actively seek the self-governance necessary to integrate a demanding professional identity with predictable family and personal life. Anchoring our findings within SDT reveals that the shifting preferences of medical graduates are not merely superficial adjustments to working conditions, but represent fundamental psychological needs that the current medical training environment must address to ensure long-term workforce engagement and prevent systemic attrition.

### Sex-related differences

In contrast, although some historical studies report marked gender differences in lifestyle and career development motivations [15], our findings confirm a growing body of recent literature showing that these gender gaps are progressively narrowing or disappearing entirely [14, 17]. The absence of sex-based differences in lifestyle considerations (F3) in our study aligns with a contemporary shifting paradigm where work–life balance has become a critical priority for both male and female trainees alike.

Several factors may explain this evolving trend, particularly among men. Generationally, modern male physicians increasingly prioritize active parenthood, equal sharing of domestic responsibilities, and the prevention of professional burnout, moving away from traditional single-earner, career-centric models. In the Israeli context, this shift is further amplified by the demographic profile of our trainees. Israeli medical students and interns are usually older than trainees in other Western countries and are more likely to have established families with children. For example, over 30% of our cohort had dependent children, compared with fewer than 4% in Australia [16]. When women and men are at comparable life stages and family status, the shared desire for predictable schedules and family involvement naturally converges, reducing the gender differences reported in younger, unmarried student populations.

### Educational stage difference

Graduates placed greater emphasis on career and academic development than last-year students, likely reflecting the shift in priorities as trainees approach residency [15]. Israeli studies show that interns report stronger research interest and feel better prepared for academic activities than earlier-stage students [16, 17], suggesting that clinical experience increases the relevance of advancement-related factors. Increased competitiveness near residency application may further reinforce research-oriented behavior, particularly in Israel’s demanding residency environment [18, 19].

### Medical school location differences

Foreign-trained graduates placed greater importance on career and academic development. This aligns with evidence that international medical graduates (IMGs) often encounter limited recognition and fewer opportunities for advancement in many health systems [20]. The Yatziv reform, which tightened Israel’s accreditation criteria for foreign medical schools, may incentivize foreign-trained graduates to demonstrate academic capabilities. At the same time, foreign-trained graduates also placed greater weight on lifestyle considerations. Internationally, IMGs often emphasize lifestyle due to migration-related challenges [21], but the Israeli context is different. Most are Israeli citizens returning from high-tuition European programs to a system with substantially longer working hours than those permitted under the European Working Time Directive [22]. When combined with Israel’s high cost of living, these factors may heighten the importance of predictable schedules and financial stability.

### Specialty-based difference

Interpersonal Factors (F1) did not differ across intended specialties, likely reflecting motivations common to the medical profession [23]. The inclusion of teaching, a professional aspiration, within this factor aligns with previous Israeli findings [24], yet this pattern may also suggest that finer distinctions within interpersonal motivations exist but did not emerge as a separate latent factor in the final model.

Participants intending surgical careers assigned the highest importance to Career and Academic Development (F2). This emphasis may reflect either the characteristics of students drawn to surgery or expectations shaped by the field. High-achieving students often prioritize opportunities for advancement [25], and research productivity is strongly linked to success in matching into surgical residencies [26]. Together, these factors suggest that interest in surgery is associated with both intrinsic motivation for academic development and awareness of its role in the residency selection process.

By contrast, those intending pediatrics or psychiatry showed strong lifestyle preferences, high interpersonal motivation, and low emphasis on career and academic development, a pattern previously described in Israeli clustering studies [27]. Specifically, candidates for Psychiatry prioritized lifestyle factors significantly more than their peers in Surgical specialties (OR = 2.62, 95% CI 1.26–5.46, *p* = 0.034). Our results place this pattern within a three-dimensional motivational framework, showing how these specialties differ specifically in lifestyle- versus career- and academic-oriented motivations. This may not reflect all future pediatricians, as students aiming for pediatric subspecialties place greater importance on prestige, income and research opportunities than those pursuing general pediatrics [28]. In Israel, although the number of pediatricians has increased, the child population has grown even faster, leaving community pediatrics in persistent shortage and undervalued relative to hospital-based work, with roles perceived as less prestigious, offering fewer opportunities for research, teaching and academic advancement [29]. These structural features are consistent with the motivational profile observed in our cohort.

### Policy implications

Our findings suggest several implications for Israeli health workforce policy. The shared importance of lifestyle considerations across sexes indicates that improving work–life balance is likely to support recruitment across multiple specialties, particularly those experiencing shortages. Accordingly, the Ministry of Health and hospital leadership should prioritize modernization of on-call systems, regulation of working hours, and development of flexible scheduling models. While implementing such structural changes in a system plagued by workforce shortages may appear counterintuitive or logistically challenging, we argue that these measures are essential for long-term system sustainability. In a shortage environment, relying on the over-extension of the existing workforce creates a compounding crisis, driving burnout, early retirement, and ‘voting with the feet’ toward alternative sectors like digital health. Therefore, improving work–life balance should not be viewed as a luxury, but as a critical retention strategy. To achieve feasibility without compromising clinical coverage, healthcare leaders must shift from simple workforce inflation to operational optimization. This includes the strategic redistribution of non-clinical administrative burdens to support staff, the implementation of predictive roster technologies, and the institutionalization of flexible part-time or shared-position models that keep physicians in the public workforce who would otherwise leave entirely.

### Strengths

A major strength of this study is the application of a robust ordinal statistical framework. Unlike previous research in this field that often treated Likert scale data as continuous variables, we utilized polychoric correlations and proportional-odds models. Recent methodological evidence demonstrates that these ordinal methods provide higher statistical power and lower bias than traditional approaches [30].

### Limitations

This study has several limitations that should be considered when interpreting the findings. First, three questionnaire items (occupational risks, difficulty coping with death, interest in specific disease) did not load on any factor, and four retained items showed relatively low communalities (between 0.23 and 0.5). These two findings suggest that additional latent factors may exist and could be identified by expanding or refining the item set in future studies. Second, respondents may differ systematically from non-respondents, such as being generally more motivated, introducing a potential selection bias. Although the study offers a comprehensive view of specialty preferences through factor analysis, it reflects the characteristics of Israeli medical trainees, who are generally older and more likely to have families than trainees in many other countries.

In addition, the study reflects preferences formed within a publicly funded health system, where residency structure, workload and career pathways are broadly influenced by governmental budgets. In this context, the way lifestyle, career and academic development considerations apply within each specialty may differ from patterns seen in countries with market driven or mixed financing models.

The questionnaire was administered only in Hebrew, which may have reduced accessibility for some foreign medical graduates. Although Hebrew proficiency is required for initiation of internship in Israel and for routine clinical work, limiting the survey to a single language may have contributed to under-representation of certain groups. In addition, recruitment relied on Israeli-based trainee networks, and we did not conduct targeted outreach to interns in peripheral hospitals or to students studying abroad who were not connected to these networks. Consequently, while graduates of Israeli medical schools represent 73% of this sample, they comprise less than half of the national intern population and thus these findings should be viewed as an exploratory baseline. Our results identify significant motivational trends that warrant further confirmation in a sample that more accurately represents the diverse educational backgrounds of the entire Israeli physician workforce.

At the time of data collection and analysis, no publicly available national data reported the sex distribution of Israeli interns or internship lottery participants, precluding assessment of representativeness for this variable.

## Conclusions

The findings in this study indicate that in contemporary generations of physicians, the need for work–life balance can no longer be overlooked during residency training. Failure to incorporate this consideration into subspecialty structures and on-call systems may ultimately result in disengagement and attrition, as trainees increasingly ‘vote with their feet transitioning into roles within medical technology and digital health startups or staying abroad. In parallel, the varying specialty specific patterns observed for career and academic development underscore the potential value of structured exposure during medical school rotations to research activity and academic career pathways in specialties traditionally perceived as less career oriented, as a strategy to strengthen recruitment to specialties in distress. In Israel, as part of residency training across all primary medical specialties, residents are required to complete a mandatory six-month research period. During this time, residents dedicate the majority of their weekly schedule to research activities, which may include wet-lab research, clinical research, or participation in research conducted within healthcare innovation or startup environments. In recent years, increasing physician workforce shortages, together with the observation that many physicians ultimately do not pursue sustained research careers, have raised questions regarding the necessity of maintaining this mandatory research period. At the core of this debate lies the need to balance strong clinical training with protected academic and research time during residency, in a way that maintains clinical competency while also fostering academic development, innovation, and long-term physician engagement in research. These issues are currently being extensively discussed by the Israeli Medical Association, the Ministry of Health, and the Ministry of Finance as part of broader deliberations on workforce planning and residency structure reform.

## Supplementary Information

Below is the link to the electronic supplementary material.

Supplementary Material 1

Supplementary Material 2

## Data Availability

The data that support the findings of this study are available from the corresponding author upon reasonable request.
