# Supplementary material for: Key factors influencing medical residency choice among medical graduates
Source: Isr J Health Policy Res. 2026 Jun 17;15:25. doi: 10.1186/s13584-026-00768-x (PMC13274235; doi:10.1186/s13584-026-00768-x)
Supplement: Supplementary file 1 — Supplementary Material 1 [file 13584_2026_768_MOESM1_ESM.docx]

**Supplementary material 1. Study questionnaire (translated from Hebrew)**

**Student/Intern Research Questionnaire**

Hello,
As part of a study being conducted at Sheba Medical Center, we aim to better understand the considerations involved in choosing a medical specialty. Therefore, we are conducting research among Israeli medical students in their final year of studies, both in Israel and abroad, as well as interns, to examine various factors related to specialty choice and perceptions of different medical fields.

This questionnaire is anonymous unless you choose to provide your personal details at the beginning. All information will be kept confidential and used solely for research purposes.

Among those who provide their details, 3 tablets will be raffled.

Estimated time to complete the questionnaire: approximately 5 minutes.

The questionnaire is worded in general language (Hebrew) and is aimed at both men and women.

**I agree to participate in the study (yes/no).**

**I agree to fill in the personal details (yes/no).**

**Full name:**

**Sex:**

- Male
- Female

**Year of Birth:**

**E-mail:**

**Phone number:**

**Academic institution-**

- Ariel University
- Ben-Gurion University
- Bar Ilan University - Safed - four-year program
- Bar Ilan University - Safed - three-year program
- Tel Aviv University - four-year program
- Tel Aviv University - six-year program
- The Hebrew University
- Haifa Technion
- Ariel University
- Abroad (joint program with Israeli hospital)
- Abroad only

**Study year:**

- 6^th^ year
- 4^th^ year in the 4-year program / 3^rd^ year in the 3-year program
- Intern
- Graduated and haven’t started my internship yet

**“Tzameret” program: (Military program)**

- Yes
- No

**Marital status**

- Single
- Married or in long-term relationship
- Divorced
- Widowed

**Economic background:**

- Below average
- Average
- Above average

**City of residence during high school:**

**Number of children:**

- **0**
- **1**
- **2**
- **3**
- **4**
- **5**
- **6-10**

**Population group and religion:**

- Jews
- Arabs – Muslims
- Circassians
- Arabs – Christians
- Armenians
- Bedouins
- Druze
- Non-Arab Christians
- Members of other religions
- Unclassified religion

**Country of birth:**

**A hospital north of Hillel Yaffe (not including) and not in Haifa, or south of Assuta Ashdod (not including), was among the top five places I ranked in the internship lottery:**

- Yes
- No
- Don’t remember
- I have not yet participated in the lottery

**Place of internship:**

- Assuta Ashdod
- Bnei Zion
- Barzilai
- Hadassah
- Carmel
- Hillel Yaffe
- Emek Medical Center
- Wolfson
- Ziv (Safed)
- Yoseftal
- Laniado
- Meir
- Maayanei Hayeshua
- Nahariya
- Nazareth
- Sourasky (Ichilov)
- Soroka
- Poriya
- Kaplan
- Rabin – Sharon
- Rabin – Beilinson
- Rambam
- Sheba
- Shamir
- Shaare Zedek
- I have not yet participated in the lottery

**Internship starting date:**

**Do you intend to pursue residency in a hospital north of Hillel Yaffe (not including) and not in Haifa, or south of Assuta Ashdod (not including)?**

- 1 – Definitely not
- 2 – Probably not
- 3 – Neutral
- 4 – Probably yes
- 5 – Definitely yes

**At this stage, in which specialty would you like to train in the future?**

- Oncology
- Pathology
- Public Health
- Geriatrics
- Anesthesiology
- Obstetrics and Gynecology
- Urology
- Orthopedic Surgery
- General Surgery
- Plastic Surgery
- Thoracic Surgery
- Pediatric Surgery
- Vascular Surgery
- ENT
- Dermatology and Venereology
- Ophthalmology
- Clinical Microbiology
- Clinical Laboratory Professions
- Neurosurgery
- Neurology
- Psychiatry (Child/Adult)
- Radiology
- Nuclear Medicine
- Emergency Medicine
- Forensic Medicine
- Rehabilitation and Physical Medicine
- Internal Medicine
- Occupational Medicine
- Pediatrics
- Family Medicine
- I do not want to specialize after internship
- I don’t know yet

**If you do not wish to specialize in medicine after completing your studies, what would you like to do?**

- 1. Biotech/pharmaceutical industry
  2. High-tech industry (for example, founding a startup)
  3. Studying another profession
  4. Working in a different field

**In the following questions, you will be asked to rate different factors based on how important they are in choosing a residency specialty. The rating is from 1–5, where:**

- 1 – Not important at all
- 2 – Not important
- 3 – Neutral
- 4 – Important
- 5 – Very important

**Factors:**

- Promotion opportunities
- Interest in procedures or specific techniques in the specialty
- Interest in the organ system or diseases related to the specialty
- Income potential
- Work–family balance
- Ability to work in the private sector
- Direct patient care
- Work environment
- Working hours
- Research opportunities
- Technology integration
- Academic promotion
- Difficulty coping with death
- Sense of purpose
- Teamwork
- High quality teaching
- Amount and quality of communication with patients
- Occupational risks (for example, radiation)
- Difficulty getting residency position

**Select the experiences that influenced your choice of residency specialty (you may choose multiple options):**

- Clerkship experience during medical school
- Personal experience as a patient / family member / acquaintance of a patient
- Acquaintances (friends/family) working in the specialty
- A physician who served as a role model
- Work experience as a physician assistant
- Research in the field
- Professional counseling
- Working in the department during internship

**In the next section, please rate the following residency specialties:**
The rating is from 1 to 5, where:
1 – Strongly disagree
2 – Disagree
3 – Neutral
4 – Agree
5 – Strongly agree

**A specialty with adequate earning potential**

- Family Medicine
- Pediatrics
- Internal Medicine
- Obstetrics and Gynecology
- General Surgery
- Orthopedics
- Plastic Surgery
- ENT
- Ophthalmology
- Psychiatry

**A specialty with strong opportunities for personal professional advancement**

- Family Medicine
- Pediatrics
- Internal Medicine
- Obstetrics and Gynecology
- General Surgery
- Orthopedics
- Plastic Surgery
- ENT
- Ophthalmology
- Psychiatry

**A specialty in which one can have a meaningful impact on a patient’s life**

- Family Medicine
- Pediatrics
- Internal Medicine
- Obstetrics and Gynecology
- General Surgery
- Orthopedics
- Plastic Surgery
- ENT
- Ophthalmology
- Psychiatry

**A specialty with a pleasant work environment**

- Family Medicine
- Pediatrics
- Internal Medicine
- Obstetrics and Gynecology
- General Surgery
- Orthopedics
- Plastic Surgery
- ENT
- Ophthalmology
- Psychiatry

**Among the specialties listed in the question, which specialty/specialties allow the best balance between work and family, etc.? (Please choose one or more).**

(Please choose one or more):

- Family Medicine
- Pediatrics
- Internal Medicine
- Obstetrics and Gynecology
- General Surgery
- Orthopedics
- Plastic Surgery
- ENT
- Ophthalmology
- Psychiatry
