# Supplementary material for: Key factors influencing medical residency choice among medical graduates
Source: Isr J Health Policy Res. 2026 Jun 17;15:25. doi: 10.1186/s13584-026-00768-x (PMC13274235; doi:10.1186/s13584-026-00768-x)
Supplement: Supplementary file 2 — Supplementary Material 2 [file 13584_2026_768_MOESM2_ESM.docx]

**Supplementary Material 2: Specialty group classification for logistic regression**

**Internal specialties**

- Internal Medicine
- Oncology
- Geriatrics
- Emergency Medicine
- Neurology

**Surgical specialties**

- Orthopedics
- Cardiothoracic Surgery
- General Surgery
- Plastic Surgery
- Otorhinolaryngology (ENT)
- Pediatric Surgery
- Urology
- Neurosurgery
- Ophthalmology

**Standalone specialty groups**

- Pediatrics
- Psychiatry (Adult/Child)
- Obstetrics and Gynecology
